# Resection of the Primary Tumor Improves the Survival of Patients With Stage IV Gastric Neuroendocrine Carcinoma

**DOI:** 10.3389/fonc.2022.930491

**Published:** 2022-07-14

**Authors:** Zefeng Li, Hu Ren, Tongbo Wang, Xiaojie Zhang, Lulu Zhao, Chongyuan Sun, Penghui Niu, Chunguang Guo, Yingtai Chen, Dongbing Zhao

**Affiliations:** Department of Pancreatic and Gastric Surgical Oncology, National Cancer Center/National Clinical Research Center for Cancer/Cancer Hospital, Chinese Academy of Medical Sciences and Peking Union Medical College, Beijing, China

**Keywords:** gastric neuroendocrine carcinoma, distant metastases, surgery, primary tumor resection, stage IV

## Abstract

**Background:**

The prognostic prolongation effect of surgical resection in the management of gastric neuroendocrine carcinoma (GNEC) with distant metastases was still uncertain. The purpose of this study was to investigate the association of primary tumor resection (PTR) with outcomes in patients with stage IV GNEC.

**Methods:**

This retrospective study analyzed patients with distant metastatic GNEC diagnosed between 2000 and 2018 and identified using the Surveillance, Epidemiology, and End Results (SEER) database. Patients were divided into PTR and non-PTR groups. The stabilized inverse probability of treatment weighting (IPTW) method was used to reduce the selection bias. Overall survival (OS) and cancer-specific survival (CSS) were estimated using the Kaplan–Meier method and log-rank test. Cox-regression analyses (uni- and multivariate) were performed to evaluate factors potentially influencing survival.

**Results:**

A total of 126 patients with a median follow-up of 79 months were identified. Forty-four patients underwent PTR and 82 patients did not undergo surgery. After the IPTW approach, PTR improved the OS in patients with stage IV GNEC (median OS 12 vs. 6 months, P = 0.010). The 1- and 3-year OS for patients with or without PTR were 43.8% and 34.5%, and 27.9% and 6.5%, respectively. The median CSS was 12 months for patients undergoing PTR and 6 months for those who did not. The 1 and 3-year CSS for patients with or without PTR were 45.1% and 37.0%, and 27.9% and 6.5%, respectively. In IPTW-adjusted Cox proportional hazards regression analysis, PTR was recognized as an independent factor for improved survival after the occurrence of distant metastatic disease [OS: hazard ratio (HR) = 0.305; 95% confidence interval (CI): 0.196, 0.475; and CSS: HR = 0.278; 95% CI: 0.171, 0.452].

**Conclusion:**

PTR for stage IV GNEC contributes to a better prognosis compared with non-surgery. This study supported the resection of the primary tumor in patients with distant metastatic GNEC.

## Introduction

Gastric neuroendocrine carcinoma (GNEC) is a rare malignant disease but with an increasing incidence in the past decades (1), constituting a spectrum of aggressive gastric malignancies (2). About a half of patients with GNEC were found to have distant metastasis at the time of diagnosis, with a relatively poor prognosis (3). In the stage IV GNEC, the national comprehensive cancer network (NCCN) suggested that chemotherapy was the first-line treatment for distant metastatic poorly differentiated neuroendocrine carcinoma, and if progression, nivolumab plus ipilimumab were considered while never mentioning the surgery (4). The standard treatment of GNEC with stage IV disease, however, has not been fully established, which makes it confusing to surgeons if the primary tumor resection (PTR) is necessary for this situation (5).

Whether palliative removal of the primary tumor can result in a survival benefit for stage IV GNEC was controversial in the previous studies (6–10). Some studies have reported the advantages of the option of PTR (7, 8), whereas others suggested not (9, 10). Pommergaard et al. (7) suggested that highly selected stage IV high-grade gastroenteropancreatic neuroendocrine and mixed neuroendocrine–non- neuroendocrine neoplasms may also benefit from surgery, but this study only contained seven GNEC. Lewis et al. (8) suggested that even patients with poorly differentiated GI-NEN may benefit from PTR in the distant metastatic setting, with or without liver treatment. However, Tierney et al. (9) and Zheng et al. (10) recommended that surgeons should perhaps refrain from resecting the primary tumor in patients with stage IV GNEC. The above studies only consisted of a small sample size of GNEC, and there was no research specializing in stage IV GNEC. As such, we conducted the research to analyze the clinicopathologic of stage IV GNEC and determine the role of PTR in distant metastatic GNEC.

## Method

### Data Collection

Surveillance, Epidemiology, and End Results (SEER) database (www.seer.cancer.gov) was used to conduct a retrospective review of patients with GNEC. The cases of GNEC were screened using the database “SEER Research Plus Data, 18 Registries, Nov 2020 Sub (2000–2018)”. The SEER database is a national database that comprises 28% of the US population and includes demographic (age, sex, and race/ethnicity), diagnostic (tumor site, tumor size, and tumor stage), treatment (surgery, radiation, and chemotherapy), and follow-up information. The data in this study were obtained from the SEER database in accordance with the SEER data use agreement (ID: 17851-Nov2020).

### Study Population

**Figure 1:**
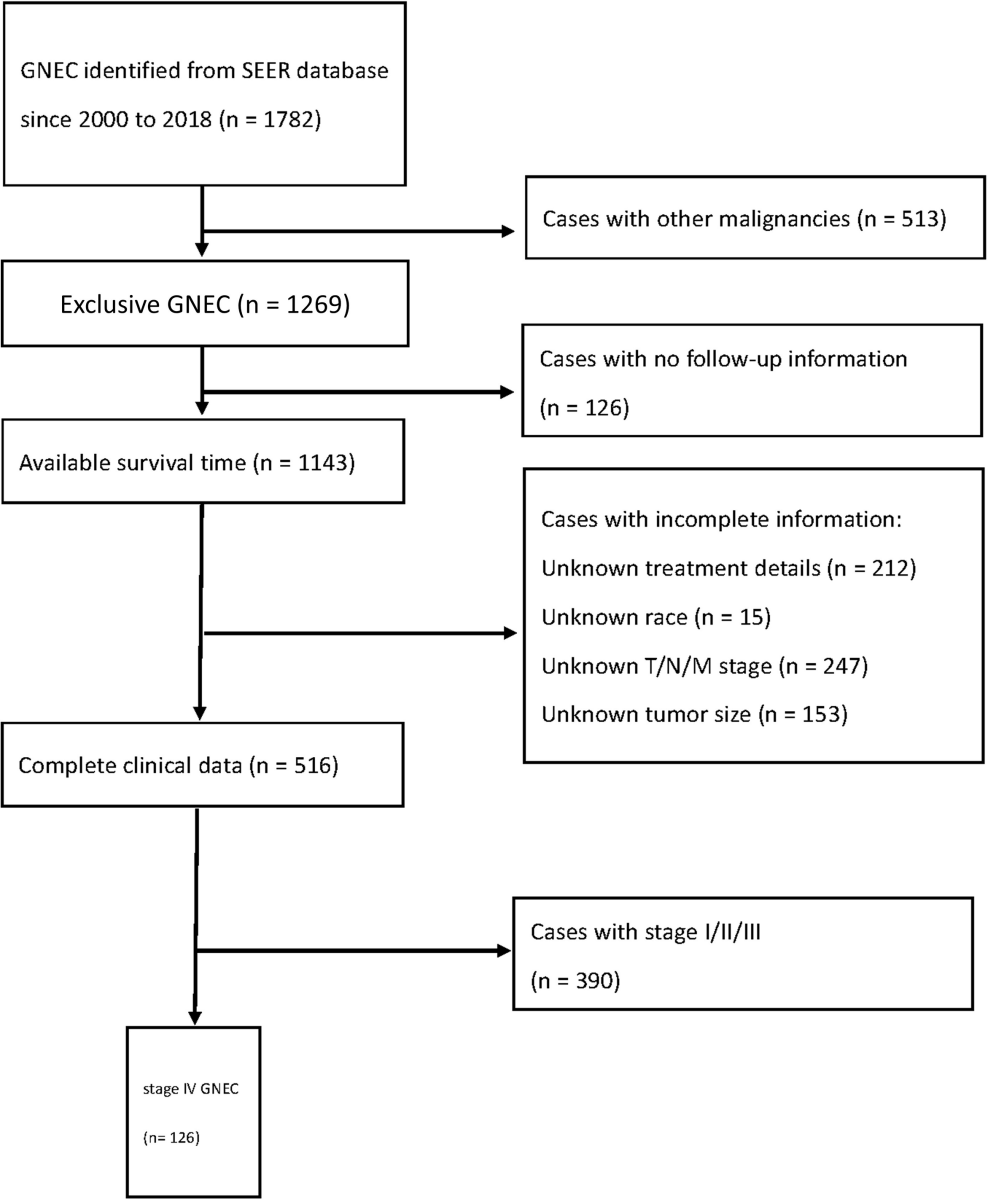
Schematic overview for patients identification.

Patients were identified according to ICD-0-3 histology codes for neuroendocrine carcinoma as “8013 (Large cell neuroendocrine carcinoma), 8041 (Small cell carcinoma, NOS), 8246 (Neuroendocrine carcinoma, NOS)”. Site-specific codes were used to identify GNEC as “C16.0–C16.9, stomach”. Patients with histologically confirmed stage IV GNEC were incorporated into the study. The exclusion criteria were cases with other malignancies, cases without follow-up information, cases with unknown treatment details, cases with unknown race, cases with unknown T/N/M stage, and cases with unknown tumor size. TNM stage was reevaluated according to the eighth edition of AJCC staging definition for gastric cancer. We only divided patients into with or without local lymph node metastasis, because many patients did not receive surgery which might contribute to a bias in the N stage. Finally, 126 patients with stage IV GNEC were recognized (**Figure 1**). Overall survival (OS) and cancer-specific survival (CSS) were defined from the date of diagnosis to the time of death or last follow-up visit.

### Statistical Analysis

Continuous variables were presented as mean [standardized difference (SD)]. Categorical variables were presented as numbers and percentages. Patient demographic characteristics (sex, race, and age), diagnostic variables (tumor site, tumor size, T stage, and local lymph node metastatic status), and treatment variables (chemotherapy, radiotherapy, and PTR) were assessed across groups using the Chi-square test for categorical nominal data and T test for continuous data. To account for the selection bias, we used the stabilized inverse probability of treatment weighting (IPTW) method to adjust the observed differences in baseline covariates between the PTR and non-PTR groups. The IPTW approach is trying to simulate a situation, in which the PTR is randomly allocated to individuals (11). A propensity score for each patient was calculated as the predicted probability of PTR from multivariable logistic regression. Factors associated either with the receipt of PTR or with survival were included in constructing the models, which included age, sex, race, tumor location, tumor size, T stage, local lymph node metastatic status, chemotherapy received, radiotherapy received, and distant oligometastasis or not. CSS and OS were compared using the log-rank test and illustrated with Kaplan–Meier curves. Multivariate analyses using the Cox proportional hazards model [included variable with a hazard ratio (HR) < 0.1 in the univariate analyses] were constructed to identify factors independently associated with prognosis. HR and 95% confidence interval (CI) were used to estimate survival predictors. We used R version 4.0.4 (R Project for Statistical Computing) and SPSS (version 23; IBM Corp, Armonk, NY, USA) to conduct the statistical analysis. Differences with an alpha level of <0.05 were considered statistically significant.

## Result

### Patient Characteristic

Demographic data of the patients with stage IV GNEC are shown in **Table 1**. A total of 126 patients were eligible for analysis. The median follow-up was 79 months, and there were 100 (79.4%) deaths recorded during the follow-up period. The mean age at diagnosis was 62.9 years old. The male-to-female ratio was 1.47:1 among these patients. Fifty-five (43.7%) patients arose in the upper third of the stomach. Fifty-two (41.3%) patients had more than one distant metastasis. The most common distant metastatic site was the ovary/peritoneum (64.3%) followed by the liver (35.7%) and distant lymph node (31.0%). Fifty-eight patients (46.0%) received perioperative or palliative chemotherapy. The 1- and 3-year OS and CSS were 40.7% and 17.5%, and 41.1%, 18.3%, respectively. PTR and non-resection groups are compared in **Table 2**. Forty-four patients (34.9%) underwent PTR and 16 of them received PTR in continuity with the resection of other organs. Specific types of the surgery performed were shown in the **Supplementary Table 1**. Patients who did not undergo resection were more likely to have a proximal GNEC than the PTR group (58.5% vs. 15.9%, P < 0.001). Chemotherapy was more common in the non-PTR group (64.6% vs. 34.1%, P = 0.002). For those who received both chemotherapy and PTR, 4 of 15 patients received chemotherapy before PTR; 9 of 15 patients received chemotherapy after PTR; 5 of 15 patients had unknown detail about the order between chemotherapy and PTR. In addition, there were no significant differences in age, sex, race, tumor size, T stage, local metastatic lymph node, distant oligometastasis or not, and radiotherapy received between patients with or without PTR. After the IPTW method, the observed differences in baseline covariates between PTR and non-PTR groups were adjusted (**Table 2**), p for all characteristics >0.1, indicating that the weighted population in the two groups was subsequently comparable.

**Table 1:** Baseline clinicopathologic characteristics of patients with stage IV gastric neuroendocrine carcinoma (N = 126, %).

**Characteristic**	
**Age, mean, SD, years**	62.9 (14.3)
**Sex**	
Men	75 (59.5)
Women	51 (40.5)
**Race**	
White	101 (80.2)
East Asian	9 (7.1)
Black	16 (12.7)
**Tumor location**	
Proximal	55 (43.7)
Middle	27 (21.4)
Distal	22 (17.5)
Mix	9 (7.1)
Unknown	13 (10.3)
**Tumor size, mean, SD, cm**	5.8 (3.6)
**T stage**	
1	22 (17.5)
2	28 (22.2)
3	28 (22.2)
4a	15 (11.9)
4b	33 (26.2)
**Local lymph node metastasis**	
No	53 (42.1)
Yes	73 (57.9)
**Surgery**	
No	82 (65.1)
Yes	44 (34.9)
**Receiving chemotherapy**	
No	58 (46.0)
Yes	68 (54.0)
**Receiving radiotherapy**	
No	111 (88.1)
Yes	15 (11.9)
**Distant metastatic number**	
More than 1	52 (41.3)
1	74 (58.7)
**Distant metastatic sites**	
Ovary/peritoneum	81 (64.3)
Liver	45 (35.7)
Distant lymph node	39 (31.0)
Bone	12 (9.5)
Lung	9 (7.1)
Brain	4 (3.2)
Unknown	3 (2.4)
**OS (95% CI)**	
1-year	40.7 (32.1, 49.3)
3-year	17.5 (10.4, 24.6)
**Median OS (95% CI), months**	11.0 (9.6, 12.4)
**CSS (95% CI)**	
1-year	41.1 (33.8, 49.7)
3-year	18.3 (11.0, 25.6)
**Median CSS (95% CI), months**	11.0 (9.6, 12.4)

**Table 2:** Baseline clinicopathologic characteristics of patients with stage IV gastric neuroendocrine carcinoma with or without primary tumor resection.

Characteristic	Unweighted Study Population, No. (%)			Weighted Study Population, No. (%)		
	No Resection N = 82	Resection N = 44	P	No Resection N = 81.05	Resection N = 46.74	P
**Age, mean, SD, years**	61.7 (14.37)	65.14 (14.05)	0.201	63.9 (14.24)	66.7 (14.64)	0.518
**Sex**			1.000			0.474
Man	49 (59.8)	26 (59.1)		49.1	32.6	
Women	33 (40.2)	18 (40.9)		32.0	14.2	
**Race**			0.788			0.302
White	67 (81.7)	34 (77.3)		63.7	27.8	
East Asian	5 (6.1)	4 (9.1)		7.1	4.0	
Black	10 (12.2)	6 (13.6)		10.3	14.9	
**Tumor location**			<0.001			0.962
Proximal	48 (58.5)	7 (15.9)		35.7	22.8	
Middle	16 (19.5)	11 (25.0)		17.6	8.7	
Distal	8 (9.8)	14 (31.8)		13.8	8.3	
Mix	4 (4.9)	5 (11.4)		4.2	2.8	
Unknown	6 (7.3)	7 (15.9)		9.8	4.2	
**Tumor size, mean, SD, cm**	5.56 (3.32)	6.18 (4.12)	0.356	6.09 (3.90)	5.98 (3.33)	0.885
**T stage**			0.409			0.806
1	15 (18.3)	7 (15.9)		13.0	7.3	
2	20 (24.4)	8 (18.2)		16.2	5.7	
3	15 (18.3)	13 (29.5)		15.9	8.4	
4a	8 (9.8)	7 (15.9)		9.6	4.6	
4b	24 (29.3)	9 (20.5)		26.3	20.8	
**Local lymph node metastasis**			0.255			0.561
No	38 (46.3)	15 (34.1)		33.7	23.9	
Yes	44 (53.7)	29 (65.9)		47.3	22.9	
**Receiving chemotherapy**			0.002			0.739
No	29 (35.4)	29 (65.9)		39.0	20.1	
Yes	53 (64.6)	15 (34.1)		42.0	26.6	
**Receiving radiotherapy**			0.114			0.272
No	69 (84.1)	42 (95.5)		71.4	2.3	
Yes	13 (15.9)	2 (4.5)		9.6	20.1	
**Distant metastatic number**			0.077			0.133
More than 1	39 (47.6)	13 (29.5)		34.9	11.5	
1	43 (52.4)	31 (70.5)		46.2	35.3	
**OS (95% CI)**						
1-year	31.2 (22.6, 43.2)	58.4 (45.4, 75.2)		27.9 (19.4, 40.2)	43.8 (23.3, 82.5)	
3-year	6.6 (2.7, 16.1)	37.7 (25.3, 56.3)		6.5 (2.6, 16.3)	34.5 (17.0, 70.1)	
**Median OS (95% CI), months**	9.0 (6.0, 11.0)	18.0 (12.0, 55.0)		6.0 (3.0, 10.0)	12.0 (12.0, 49.0)	
**CSS (95% CI)**						
1-year	31.2 (22.6, 43.2)	60.1 (47.0, 76.8)		27.9 (19.4, 40.2)	45.1 (23.7, 85.8)	
3-year	6.6 (2.7, 16.1)	40.9 (27.9, 60.0)		6.5 (2.6, 16.3)	37.0 (18.3, 74.8)	
**Median CSS (95% CI), months**	9.0 (6.0, 11.0)	27.0 (12.0, NA)		6.0 (3.0, 10.0)	12.0 (12.0, NA)	

### Survival Analysis

**Figure 2:**
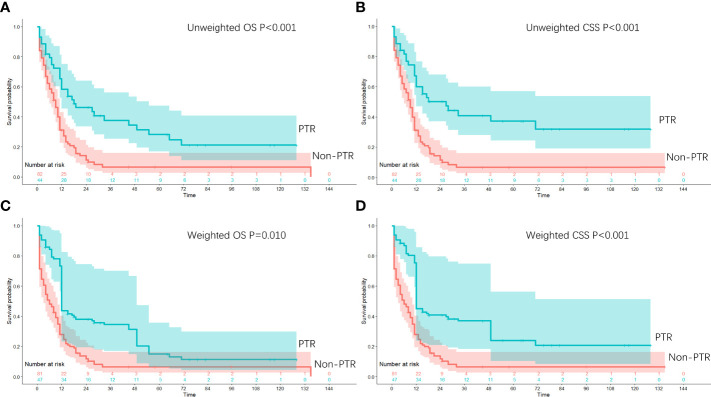
Kaplan–Meier survival curves for patients with stage IV gastric neuroendocrine carcinoma with or without primary tumor resection in the unweighted study population (**A**: OS; **B**: CSS) and in the weighted study population (**C**: OS; **D**: CSS).

In the weighted population, patients who underwent PTR had a median OS of 12.0 months versus a median OS of 6.0 months among patients who did not undergo PTR (P = 0.010, **Table 2**), with 1- and 3-year OS of 43.8% and 34.5%, and 27.9% and 6.5%, respectively. Median CSS was 12.0 months for the patients who underwent PTR and 6.0 months for patients who did not undergo resection (P < 0.001, **Table 2**), with 1- and 3-year CSS of 45.1% and 37.0%, and 27.9%, 6.5%, respectively. Kaplan–Meier survival curves of OS and CSS (before and after the IPTW method) were shown based on PTR receiving status (**Figure 2**).

To further verified the result, in the multivariable analysis, PTR remained independently associated with prolonged OS (HR = 0.305; 95% CI: 0.196–0.475) and CSS (HR = 0.278; 95% CI: 0.171–0.452). Negative predictors of OS identified on multivariate cox analyses included older age, male, larger tumor size, and lack of PTR and chemotherapy (**Table 3**). The factors associated with worse CSS in multivariable analysis were increasing age, male, larger tumor size, and lack of PTR and chemotherapy (**Table 4**).

**Table 3:** Univariable and multivariable cox regression analyses of factors associated with overall survival of patients with stage IV gastric neuroendocrine carcinoma in the Weighted Study Population.

Clinicopathological features	Univariable analysis		Multivariable analysis	
	HR (95% CI)	P	HR (95% CI)	P
**Age**	1.023 (1.009, 1.037)	0.001	1.027 (1.008, 1.046)	0.004
**Sex**				
Men	1 (Reference)		1 (Reference)	
Women	0.630 (0.400, 0.992)	0.046	0.475 (0.279, 0.808)	0.006
**Race**			NA	NA
White	1 (Reference)			
East Asian	1.446 (0.554, 3.769)	0.451		
Black	1.290 (0.822, 2.026)	0.268		
**Tumor location**				
Proximal	1 (Reference)		1 (Reference)	
Middle	0.962 (0.518, 1.785)	0.902	1.008 (0.502, 2.026)	0.981
Distal	0.891 (0.429, 1.851)	0.756	1.081 (0.498, 2.345)	0.844
Mix	0.511 (0.255, 1.024)	0.059	0.509 (0.187, 1.385)	0.186
Unknown	1.141 (0.529, 2.460)	0.737	0.865 (0.441, 1.700)	0.675
**Tumor size**	1.007 (1.000, 1.014)	0.048	1.007 (1.002, 1.013)	0.011
**T stage**			NA	NA
1	1 (Reference)			
2	1.088 (0.532, 2.225)	0.817		
3	1.111 (0.497, 2.487)	0.797		
4a	1.190 (0.459, 3.087)	0.721		
4b	1.260 (0.615, 2.582)	0.529		
**Local lymph node metastasis**			NA	NA
No	1 (Reference)			
Yes	1.370 (0.884, 2.124)	0.159		
**Surgery**				
No	1 (Reference)		1 (Reference)	
Yes	0.465 (0.293, 0.739)	0.001	0.305 (0.196, 0.475)	<0.001
**Receiving chemotherapy**				
No	1 (Reference)		1 (Reference)	
Yes	0.614 (0.393, 0.957)	0.031	0.530 (0.332, 0.846)	0.008
**Receiving radiotherapy**			NA	NA
No	1 (Reference)			
Yes	1.322 (0.895, 1.955)	0.161		
**Distant metastatic number**				
More than 1	1 (Reference)		NA	NA
1	0.866 (0.555, 1.353)	0.528		

NA, Not Available.

**Table 4:** Univariable and multivariable cox regression analyses of factors associated with cancer-specific survival of patients with stage IV gastric neuroendocrine carcinoma in the weighted study population.

Clinicopathological features	Univariable analysis		Multivariable analysis	
	HR (95% CI)	P	HR (95% CI)	P
**Age**	1.020 (1.006, 1.035)	0.004	1.025 (1.006, 1.045)	0.010
**Sex**				
Men	1 (Reference)		1 (Reference)	
Women	0.648 (0.405, 1.036)	0.070	0.504 (0.289, 0.879)	0.016
**Race**			NA	NA
White	1 (Reference)			
East Asian	1.131 (0.305, 4.199)	0.854		
Black	1.312 (0.837, 2.056)	0.236		
**Tumor location**				
Proximal	1 (Reference)		1 (Reference)	
Middle	0.873 (0.427, 1.787)	0.711	0.932 (0.431, 2.019)	0.859
Distal	0.922 (0.447, 1.901)	0.826	1.111 (0.500, 2.467)	0.796
Mix	0.519 (0.259, 1.037)	0.063	0.494 (0.179, 1.362)	0.173
Unknown	1.196 (0.564, 2.536)	0.642	0.878 (0.448, 1.722)	0.706
**Tumor size**	1.008 (1.001, 1.015)	0.029	1.008 (1.003, 1.014)	0.004
**T stage**			NA	NA
1	1 (Reference)			
2	0.948 (0.433, 2.077)	0.894		
3	0.976 (0.428, 2.226)	0.953		
4a	1.135 (0.438, 2.942)	0.794		
4b	1.215 (0.593, 2.489)	0.594		
**Local lymph node metastasis**			NA	NA
No	1 (Reference)			
Yes	1.408 (0.887, 2.236)	0.147		
**Surgery**				
No	1 (Reference)		1 (Reference)	
Yes	0.409 (0.245, 0.686)	<0.001	0.278 (0.171, 0.452)	<0.001
**Receiving chemotherapy**				
No	1 (Reference)		1 (Reference)	
Yes	0.650 (0.407, 1.038)	0.071	0.551 (0.342, 0.888)	0.014
**Receiving radiotherapy**			NA	NA
No	1 (Reference)			
Yes	1.349 (0.912, 1.997)	0.135		
**Distant Metastatic number**			NA	NA
More than 1	1 (Reference)			
1	0.870 (0.541, 1.395)	0.561		

NA, Not Available.

**Figure 3:**
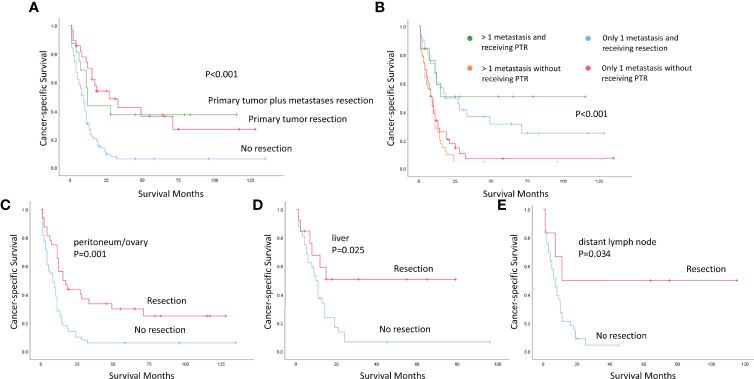
Kaplan–Meier survival curves for patients with stage IV gastric neuroendocrine carcinoma in the unweighted study population [**A**: with or without distant metastases resection; **B**: with 1 or more than 1 distant metastasis. Subgroup analysis by different distant metastatic site (**C**: peritoneum/ovary, **D**: liver, **E**: distant lymph node)]. PTR, primary tumor resection.

Furthermore, we evaluated the role of PTR in subgroups in the crude dataset. To clarify the interaction of the distant metastases resection and PTR, **Figure 3A** showed that PTR could improve the CSS no matter receiving the resection of distant metastases or not. Subgroup analysis by distant metastatic number showed a similar improvement in CSS irrespective of distant metastatic number (**Figure 3B**). PTR offered a survival benefit whether is distant oligometastatic or not. Analysis by distant metastatic site showed improved CSS in patients with peritoneum/ovary, liver, and distant lymph node metastases who underwent PTR (**Figures 3C–E**).

## Discussion

Our study provided the first comprehensive analysis of the role of PTR in stage IV GNEC with sufficient follow-up and substantial information on important variables. Significant survival benefits were observed in patients with PTR compared with those who did not.

The poor survival of patients with stage IV GNEC was observed in the present study. The median OS was 11 months, the same as the previous report about high-grade gastroenteropancreatic neuroendocrine carcinoma (7), worse than patients with gastric adenocarcinoma (12). In addition, the median CSS was 11 months. The 1- and 3-year OS and 1- and 3-year CSS were 40.7% and 17.5%, and 41.1% and 18.3%, respectively (**Table 1**). Consistent with the previous study (6), GNEC was more prone to arise in the upper third of the stomach. In addition, we found that primary tumor location was not balanced between PTR and non-PTR groups in the unweighted dataset. Tumor location of the distal stomach was associated with resection, likely because surgeries in these locations were considered easier to perform with less complications. A significant interaction was noted between treatment effect and primary tumor location (13). Moreover, in the crude data, patients, who received PTR, indeed were less likely to receive chemotherapy. It is speculated that, after receiving an operation like gastrectomy, the patients have a lower tolerance to chemotherapy’ side effect (14). The IPTW approach was attempting to mimic a situation, in which PTR was randomly allocated to individuals (15). After the IPTW method, the above confounders were successfully balanced between the two intervention groups in the weighted population.

PTR could improve the prognosis of stage IV GNEC in the weighted dataset and all subgroups. In the weighted dataset, the patients with stage IV GNEC who underwent PTR demonstrated significantly prolonged survival than those who did not (median OS/CSS, 12.0 months versus 6.0 months; OS, P = 0.010; CSS, P < 0.001; **Table 2**). The multivariate analysis further identified that age, sex, tumor size, PTR received, and chemotherapy received were the independent prognostic predictors (**Tables 3**, **4**). To further clarify the role of PTR in heterogeneous stage IV GNEC, we conducted the subgroup analysis. Chakedis et al. (16) reported that the removal of all distant metastatic diseases was associated with the longest median survival compared with debulking or palliative resection in patients with distant metastatic gastroenteropancreatic neuroendocrine tumors. In the present study, surgical treatment of the primary tumor with or without additional resection of distant metastases was both associated with a better prognosis (**Figure 3A**). The number of distant metastatic foci represented the tumor burden to some extent. We showed that PTR could improve the prognosis for patients with stage IV GNEC, regardless of the distant oligometastasis or the multi-organ metastases (**Figure 3B**). Yoshida et al. (12) proposed that patients with stage IV gastric cancer can be divided on the basis of the absence (categories 1 and 2) or presence (categories 3 and 4) of macroscopically detectable peritoneal dissemination, which has a different biological outcome compared with hematological metastasis. In our study, the peritoneum/ovary was the most common distant metastatic site. In addition, 45 patients presented with liver metastases, which was reported to be the most common site of distant metastatic gastrointestinal neuroendocrine neoplasms (17). The metastatic pattern of GNEC needs to be further investigated in the future. Significantly improved OS after gastrectomy was observed across all patient subgroups, comprising patients with peritoneal/ovary metastasis, liver metastasis, and distant lymph node metastasis (**Figures 3C–E**).

Many researchers supported PTR in patients with distant metastatic well-differentiated gastrointestinal neuroendocrine tumors, which the current guidelines from both ENETS and NCCN advocated (8, 10, 18, 19). Debulking operations are recommended for patients with distant metastatic neuroendocrine tumors, because debulking improves symptomatic control of hormone hypersecretion and survival. As for specific gastric neuroendocrine tumors, which rarely present with hormone hypersecretion, PTR was also associated with prolonged survival among patients with stage IV gastric neuroendocrine tumors who did not undergo resection of distant metastatic disease at any metastatic site (9, 17). In contrast to neuroendocrine tumors, surgery is the mainstay of treatment for GNEC without distant metastasis, whereas palliative chemotherapy remains the standard of care for distant metastatic GNEC. The chemotherapy regimens of GNEC are extrapolated from published data on small cell carcinoma of the lung and gastric adenocarcinoma. Various types of chemotherapy have been used to treat GNEC. Regimens containing cisplatin plus irinotecan were suggested to produce a good response in GNEC, with an overall response rate of 75% and a PFS of 212 days (20). Our study confirmed the critical role of chemotherapy in stage IV GNEC. However, surgery was not even mentioned in the section of the guidelines on treatment for distant metastatic GNEC (21). For colorectal neuroendocrine carcinoma, Smith et al. (22)suggested that the surgery in the presence of distant metastatic disease might not offer a survival benefit. However, Haugvik et al. (23) demonstrated that resection of the primary tumor was an independent prognostic factor of improved survival for patients with distant metastatic pancreatic neuroendocrine carcinoma. Our study revealed that PTR could prolong the survival of patients with stage IV GNEC using the SEER database.

There are two possible explanations for the prognosis-prolonging effect of PTR. First, reduction of immunosuppressive tumor burden may have extended the prognosis, potentially minimizing the chance that the tumor will lead to disease progression and further metastases. Second, it is suggested that chemotherapy compliance may be improved by PTR in symptomatic patients (13, 24). The actual mechanism of how PTR may contribute to improved outcomes remains not clearly understood. On the other hand, with the continual progress of surgical techniques, the safety of palliative gastrectomy had increased in recent years. There was no difference in postoperative complications between the groups of distal gastrectomy and gastrojejunostomy. It can be said that distal gastrectomy can be safely performed even in patients with stage IV gastric cancer (24). In situations where surgical resection would not be expected to cause significant complications, the approach of PTR as needed seems reasonable for patients with stage IV GNEC. Although the effectiveness of surgery was limited, it remains one of the most important modalities for treating GNEC. In the era of personalized treatment, PTR should be considered as a part of a comprehensive multidisciplinary approach for the optimal treatment of patients with stage IV GNEC.

Owing to the nature of the database, the current study had several limitations that should be taken into account, when interpreting the results. First, this analysis was retrospective and subject to selection bias despite conducting the IPTW method. Second, critical variables may be inadequate. Information on detailed data on performance status, presence of comorbidities, heterogeneous chemotherapy regimens, treatment on distant metastases, and the reasons why they received or did not receive PTR were absent in the SEER database. Third, for patients with a limited lifespan, the assessment of the quality of life was crucial, which was not available in this study. When choosing the optimum treatment strategy, the consideration of patients must include various factors that predict the quality of life. Quality of life should be the principal criterion in the treatment of such pathology that remains incurable at present. Goals of care should focus on both oncologic and quality-of-life impacts (25). In particular, limited to the sample size, this study lacked the granularity to identify certain subsets of patients who may derive the most benefit from surgery in the setting of stage IV disease. Despite these limitations, we believed that the comprehensive information on the clinicopathological characteristics, surgical outcome, and prognosis of GNEC was enough to support our findings that PTR was an independent prognostic factor associated with prolonged survival in GNEC. Further well-designed randomized clinical studies with longer follow-ups are warranted. Such studies should also aim to evaluate the efficacy of PTR in stage IV GNEC, both in terms of its survival impact and preservation of health-related quality of life. Until then, PTR in patients with stage IV GNEC should probably be considered after thorough multidisciplinary discussions involving the patient basis.

## Conclusion

In summary, we found that PTR significantly prolonged OS and CSS compared with non-surgery in patients with stage IV GNEC. The positive results in our study need to be replicated in larger population studies with greater power.

## Data Availability Statement

The raw data supporting the conclusions of this article will be made available by the authors, without undue reservation.

## Ethics Statement

The studies involving human participants were reviewed and approved by Institutional Review Board at the China National Cancer Center. Written informed consent for participation was not required for this study in accordance with the national legislation and the institutional requirements.

## Author Contributions

Conception and design: ZL, HR, YC, and DZ; Collection and assembly of data: ZL, LZ, CS, PN, and XZ; Data analysis and interpretation: ZL, HR, TW, and CG; Manuscript writing: All authors; Final approval of the manuscript: All authors.

## Funding

This study received funding from Beijing Hope Run Special Fund of Cancer Foundation of China (No. LC2021B20).

## Conflict of Interest

The authors declare that the research was conducted in the absence of any commercial or financial relationships that could be construed as a potential conflict of interest.

## Publisher’s Note

All claims expressed in this article are solely those of the authors and do not necessarily represent those of their affiliated organizations, or those of the publisher, the editors and the reviewers. Any product that may be evaluated in this article, or claim that may be made by its manufacturer, is not guaranteed or endorsed by the publisher.
