# Supplementary material for: Resection of the Primary Tumor Improves the Survival of Patients With Stage IV Gastric Neuroendocrine Carcinoma
Source: Front Oncol. 2022 Jul 14;12:930491. doi: 10.3389/fonc.2022.930491 (PMC9329560; doi:10.3389/fonc.2022.930491)
Supplement: Supplementary file 1 [file Table_1.docx]

Supplementary Table 1. The specific type of surgery performed in the primary tumor resection group

| Type | No. |
| --- | --- |
| Polypectomy | 3 |
| Gastrectomy, NOS (partial, subtotal, hemi-) | 7 |
| Antrectomy, lower (distal-less than 40% of stomach) | 2 |
| Lower (distal) gastrectomy (partial, subtotal, hemi-) | 6 |
| Upper (proximal) gastrectomy (partial, subtotal, hemi-) | 1 |
| Near-total gastrectomy | 1 |
| Total gastrectomy | 4 |
| Gastrectomy, NOS WITH removal of a portion of esophagus | 1 |
| Partial or subtotal gastrectomy | 3 |
| Gastrectomy with a resection in continuity with the resection of other organs, NOS | 1 |
| Partial or subtotal gastrectomy, in continuity with the resection of other organs | 9 |
| Near total or total gastrectomy, in continuity with the resection of other organs | 4 |
| Radical gastrectomy, in continuity with the resection of other organs | 2 |

NOS: Not otherwise specified.
